# Histone tail analysis reveals H3K36me2 and H4K16ac as epigenetic signatures of diffuse intrinsic pontine glioma

**DOI:** 10.1186/s13046-020-01773-x

**Published:** 2020-11-25

**Authors:** Shejuan An, Jeannie M. Camarillo, Tina Yi-Ting Huang, Daphne Li, Juliette A. Morris, Madeline A. Zoltek, Jin Qi, Mandana Behbahani, Madhuri Kambhampati, Neil L. Kelleher, Javad Nazarian, Paul M. Thomas, Amanda M. Saratsis

**Affiliations:** 1 Department of Neurological Surgery, Northwestern University Feinberg School of Medicine, Chicago, IL USA; 2 Department of Chemistry, Molecular Biosciences and Proteomics Center of Excellence, Northwestern University, Evanston, IL 60208 USA; 3 Department of Neurological Surgery, Loyola University, Chicago, IL USA; 4 Department of Neurological Surgery, University of Illinois Chicago, Chicago, IL USA; 5 Research Center for Genetic Medicine, Children’s National Health System, Washington, DC USA; 6 Department of Biochemistry and Molecular Genetics, Northwestern University Feinberg School of Medicine, Chicago, IL USA; 7 Department of Integrative Systems Biology, George Washington University School of Medicine and Health Sciences, Washington, DC USA; 8 Department of Oncology, University Children’s Hospital, Zurich, Switzerland; 9 Division of Pediatric Neurosurgery, Department of Surgery, Ann & Robert H. Lurie Children’s Hospital of Chicago, 225 E Chicago Avenue, Box 28., Chicago, IL 60611-2991 USA

**Keywords:** DIPG, Histone, H3K27M, H3K36me2, H4K16ac

## Abstract

**Background:**

Diffuse intrinsic pontine glioma (DIPG) is an aggressive pediatric brainstem tumor. Most DIPGs harbor a histone H3 mutation, which alters histone post-translational modification (PTM) states and transcription. Here, we employed quantitative proteomic analysis to elucidate the impact of the H3.3K27M mutation, as well as radiation and bromodomain inhibition (BRDi) with JQ1, on DIPG PTM profiles.

**Methods:**

We performed targeted mass spectrometry on H3.3K27M mutant and wild-type tissues (*n* = 12) and cell lines (*n* = 7).

**Results:**

We found 29.2 and 26.4% of total H3.3K27 peptides were H3.3K27M in mutant DIPG tumor cell lines and tissue specimens, respectively. Significant differences in modification states were observed in H3.3K27M specimens, including at H3K27, H3K36, and H4K16. In addition, H3.3K27me1 and H4K16ac were the most significantly distinct modifications in H3.3K27M mutant tumors, relative to wild-type. Further, H3.3K36me2 was the most abundant co-occurring modification on the H3.3K27M mutant peptide in DIPG tissue, while H4K16ac was the most acetylated residue. Radiation treatment caused changes in PTM abundance in vitro, including increased H3K9me3. JQ1 treatment resulted in increased mono- and di-methylation of H3.1K27, H3.3K27, H3.3K36 and H4K20 in vitro.

**Conclusion:**

Taken together, our findings provide insight into the effects of the H3K27M mutation on histone modification states and response to treatment, and suggest that H3K36me2 and H4K16ac may represent unique tumor epigenetic signatures for targeted DIPG therapy.

**Supplementary Information:**

The online version contains supplementary material available at 10.1186/s13046-020-01773-x.

## Background

Pediatric high-grade glioma is the leading cause of cancer death in children [1]. Diffuse intrinsic pontine glioma (DIPG) is a high-grade glioma that arises in the brainstem, with an overall survival of only 11 months [2]. DIPG cannot be removed surgically, and there is no known effective treatment. Standard management is with radiotherapy, which temporarily relieves symptoms but only confers a survival benefit of three months [3]. Since DIPGs ultimately exhibit treatment resistance and disease progression, better understanding of tumor biology is desperately needed to identify effective therapeutic therapy [4].

Recent studies suggest that changes in epigenetic regulation of gene transcription play a key role in pediatric gliomagenesis and response to treatment [5–7]. Dynamic post-translational modifications (PTMs) on the histone N-terminal tail, rendered by histone modifying enzymes, alter chromatin structure to facilitate open reading (euchromatin) or silencing (heterochromatin) throughout the genome [5, 8–10]. Specific patterns of histone PTMs represent an epigenetic signature, or histone code, reflecting a relative transcriptional state. In up to 80% of DIPGs, heterozygous somatic mutations in genes encoding histone H3 isoforms H3.1 and H3.3 (*HIST1H3B* and *H3F3A,* respectively) substitute methionine for lysine at amino acid residue 27 on the H3 N-terminal tail (H3K27M) [11]. This H3K27M mutant protein alters histone H3 PTMs in DIPG, impacting chromatin structure and hence gene expression [5, 7, 12].

For example, histone H3K27 di- and tri-methylation (H3K27me2/3), transcriptionally silencing marks, are reduced in H3K27M DIPG, de-repressing transcription [13–15]. H3K27me2/3 loss is accompanied by increased H3K27ac and the formation of heterotypic H3K27M/H3K27ac nucleosomes that colocalize with bromodomain proteins 2 and 4 (BRD2/4) and RNA polymerase II, suggesting a potential role of BRD proteins in DIPG pathogenesis [7, 16]. We demonstrated pre-clinical efficacy of bromodomain inhibition (BRDi) with JQ1, a bromodomain and extra terminal domain (BET) inhibitor, resulting in reduction of H3K27ac and restoration of H3K27me3 [7]. These findings suggest that changes in epigenetic regulation of gene expression in DIPG may be reflected by histone codes, and detection of these signatures could shed light on mechanisms of tumorigenesis. Here, we employ targeted mass spectrometry via multiple reaction monitoring (MRM) to characterize 93 distinct PTM states in H3.3K27M mutant DIPG specimens, and investigate the effects of radiation and bromodomain inhibition on tumor epigenetic signatures.

## Materials and methods

### Tissue specimens

Pediatric DIPG tumor tissue (*n* = 9, H3.3K27M mutant) was collected postmortem (IRB #Pro00001339). H3.3K27 wild-type (WT) adult glioma tumor tissue specimens (*n* = 2), and one non-tumor frontal lobe tissue specimen were collected during the course of treatment (IRB# STU00095863). All diagnoses were made radiographically by a neuroradiologist, then confirmed by neuropathological evaluation upon tissue collection. Patient identifiers were removed and a single sequential numerical identifier assigned to each specimen (Table 1). Tissues were stored at − 80 °C prior to proteomic analysis (IRB #STU00202063).

**Table 1:** Summary of specimens analyzed with histone tail mass spectrometry

ID	Specimen type	Histone Status	Gender	Age (Years)
**1**	**DIPG tissue**	**H3.3K27M**	**F**	**5**
**2**	**DIPG tissue**	**H3.3K27M**	**M**	**8**
**3**	**DIPG tissue**	**H3.3K27M**	**M**	**7**
**4**	**DIPG tissue**	**H3.3K27M**	**M**	**19.6**
**5**	**DIPG tissue**	**H3.3K27M**	**F**	**7**
**6**	**DIPG tissue**	**H3.3K27M**	**M**	**7.6**
**7**	**DIPG tissue**	**H3.3K27M**	**M**	**6**
**8**	**DIPG tissue**	**H3.3K27M**	**M**	**6**
**9**	**DIPG tissue**	**H3.3K27M**	**M**	**6.25**
**10**	**GBM tissue**	**H3.3G34R**	**N/A**	**45**
**11**	**GBM tissue**	**Wild type**	**N/A**	**50**
**12**	**Focal cortical dysplasia, frontal lobe**	**Wild type**	**N/A**	**62**
**SF7761**	**DIPG cell line**	**H3.3K27M**	**N/A**	**N/A**
**SF8628**	**DIPG cell line**	**H3.3K27M**	**N/A**	**N/A**
**DIPG 007**	**DIPG cell line**	**H3.3K27M**	**N/A**	**N/A**
**KNS 42**	**GBM cell line**	**H3.3G34V**	**N/A**	**N/A**
**SF9427**	**Pediatric GBM cell line**	**Wild type**	**N/A**	**N/A**
**U87**	**Adult GBM cell line**	**Wild type**	**N/A**	**N/A**
**NHA**	**Normal human astrocytes**	**Wild type**	**N/A**	**N/A**

### Cell lines

Patient-derived pediatric DIPG cell lines SF8628, SF7761, DIPG007 (H3.3K27M mutant) and high-grade glioma cells KNS42 (H3.3G34V mutant) and SF9427 (H3.3G34 wild-type) were provided by Dr. Rintaro Hashizume (Northwestern University) as previously described [17–19]. H3 wild-type adult glioma cells (U87) and normal human astrocytes (NHA) were obtained from American Type Culture Collection and ScienCell (#1800), respectively. Cells were treated with 300 nM JQ1 (Selleckchem S7110) or DMSO (Sigma D2650) for 24 and 48 h. Cell irradiation (9 Gy) was performed with the RadSOURCE-2000 X-Ray Irradiator, and cells analyzed at 24 and 48 h. Cell pellets were generated by harvesting with trypsin and washing twice with PBS, then stored at − 80 °C prior to proteomic analysis.

### Western blotting

Histones were extracted from cell pellets using Histone Extraction Kit (ab113476, Abcam) according to the manufacturer’s protocol. 0.5 μg Histones were loaded and separated by SDS-PAGE using a 12% Mini-PROTEAN® TGX™ Precast Protein Gels and transferred by using Trans-Blot Turbo Mini 0.2 μm PVDF Transfer Packs at 2.5A, 25 V for 3 min in a Trans-Blot Turbo™ Transfer Systems (Bio-Rad). The membrane was blocked prior to the addition of the primary antibody with 5% milk in Tris buffered saline with 0.1% of Tween 20. The membrane was incubated overnight with primary antibody in TBS buffer with 0.1% Tween and 5% milk. The dilution of primary antibody was as follows: Rabbit monoclonal anti-Histone H3 (mutated K27M) antibody (ab190631) at a dilution of 1:1000, Rabbit mAb Acetyl-Histone H3 (Lys27) (D5E4) XP ((#8173, Cell Signaling Technology) at a dilution of 1:1000, Mouse mAb Histone H3 (1B1B2) (#14269, Cell Signaling Technology) at a dilution of 1:1000. The membrane was washed 3 times with TBS/0.1% Tween and incubated with an anti-rabbit or anti-mouse IgG conjugated to horse radish peroxidase (7074 and 7076, Cell Signaling Technology) at a 1:2000 dilution in TBS/0.1% Tween and 5% milk. Pierce ECL Plus Western Blotting Substrate (CN: 32132, Thermo Scientific) was used according to the manufacturer’s protocol to visualize proteins and quantify band intensity. Experiments were performed in triplicate for statistical analysis. The intensity of protein bands were analyzed by ImageJ, with the relative protein value expressed via the ratio of specific protein/H3.

### Gene expression profiling

Total cell RNA was extracted for gene expression profiling based on manufacturer protocol (Qiagen). All samples were adjusted to a concentration of 20 ng/μl RNA prior to submission for gene expression analysis using the NanoString nCounter Plexset Analysis System DNA damage repair 96 gene codeset (NanoString Technologies, Seattle, WA). Gene expression values were determined and converted to fold change in expression level for each treatment condition compared to DMSO controls in order to determine statistically significant differences in gene expression levels between groups (NanoString). Functional pathways and gene ontology analysis was then performed on fold change expression values (Inginuity Pathways Analysis, Qiagen).

### Histone preparation for targeted mass spectrometry

Nuclei were isolated in buffer (15 mM Tris-HCl pH 7.5, 60 mM KCl, 15 mM NaCl, 5 mM MgCl_2_, 1 mM CaCl_2_, 250 mM sucrose; 1 mM DTT, 1:100 HALT protease inhibitor (Thermo Scientific)), and 10 mM sodium butyrate added immediately prior to use containing 0.3% NP-40. Nuclei were centrifuged at 600 x *g* for five minutes at 4 °C and washed twice with nuclear isolation buffer. Histones were extracted by via five volumes of H_2_SO_4_ and incubated at room temperature one hour. Debris was removed by centrifugation at 4000 x *g* for five minutes. Trichloroacetic acid was added to the supernatant at a final concentration of 20% (v/v) and incubated overnight at 4 °C. Precipitated histones were pelleted at 10,000 x *g* for five minutes, washed once with ice-cold acetone with 0.1% HCl and twice with acetone at 15,000 x *g* for five minutes. Residual acetone was dried in a fume hood, and samples stored at − 20 °C until further processing. Histones were derivatized with a single round of propionylation, digested with 1 μg trypsin (Promega, Madison, WI), and derivatized again with a single round of propionylation according to Garcia et al. [20].

### Targeted LC-MS/MS of histone peptides

Histone peptides were resuspended in Loading Buffer (0.1% TFA in water) and analyzed on a Thermo TSQ Quantiva (Thermo Scientific) in-line with a Dionex nano-LC (Sunnyvale, CA). Peptides were loaded onto a trapping column (3 cm × 150 μm, packed with ProntoSIL C18-AQ, 3 μm, 200 Å resin (New Objective, Woburn, MA)) for 10 min at 2.5 μL/min with 100% Loading Buffer. Elution from the trapping column and separation on a PicoChip analytical capillary column (10 cm × 75 μm packed with ProntoSIL C18-AQ, 3 μm, 200 Å resin (New Objective)) was achieved by decreasing the percentage of Solvent A (0.1% formic acid in water) and increasing the percentage of Solvent B (0.1% formic acid in 95% acetonitrile) from 1 to 35% at a flow rate of 0.30 μL/min over 45 min. The peptides were introduced into the triple quadrupole mass spectrometer by electrospray from an emitter with a 10 μm tip. The instrument settings were as follows: collision gas pressure of 1.5 mTorr; Q1 peak width of 0.7 (FWHM); cycle time of 3 s; skimmer offset of 10 V; electrospray voltage of 2.5 kV. All injections were performed in technical triplicate. Targeted methods were performed as previously published [21]. A list of monitored peptide transitions is provided (Supplementary material 1).

### Quantitation of histone modification states

Raw data were imported into Skyline with Savitzky-Golay smoothing for analysis. Peak area assignments were manually confirmed, and total peak areas used to determine the percent relative abundance of histone modifications. The percent relative peptide abundance was determined by dividing the area under the curve for a given peptide of interest by the sum of the areas under the curve for all peptides with the same sequence, then multiplying by 100. Heatmaps were generated with R using gplots package, and show modifications that were above the limit of detection in all samples.

### Statistical analysis

Differences in peptide abundance were analyzed using independent-sample t test for between-group comparisons, or one-way ANOVA and Tukey’s post hoc test for multi-group analyses using SPSS (IBM, Version 25), and expressed as mean ± standard deviation (SD). Pearson Correlation was performed to analyze the relationship between H3.3K27M mutation and detected histone codes. Multivariable analysis was performed using linear regression with Stepwise entry (*p* = 0.05) and removal (*p* = 0.10). All *p* values were two-sided, with *p* < 0.05 considered statistically significant.

## Results

### Glioma cell histone codes are distinct by H3.3K27M mutation status

**Fig. 1:**
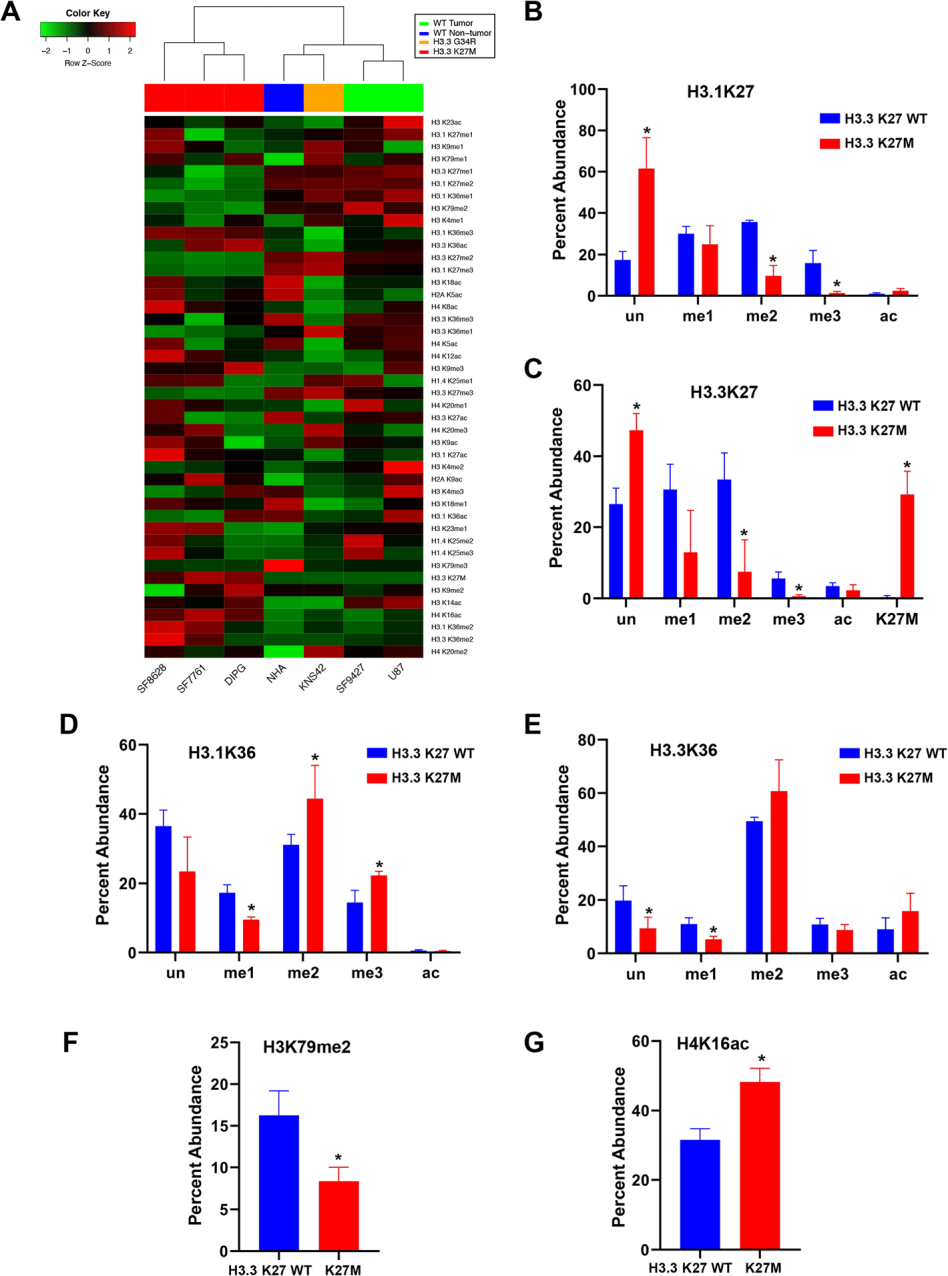
Histone peptide modification states in pediatric glioma cell lines. **a** Unsupervised hierarchical clustering of pediatric glioma cell line histone tail modification profiles reveals clustering by H3K27M mutation status. **b-e** Bar graph showing that relative abundance of modifications at H3.1K27/K36 and H3.3K27/K36 in glioma cell lines are distinct by H3.3K27M mutation status. **f-g** Statistically significant differences in H3K79me2 and H4K16ac abundance are observed in H3.3K27M mutant glioma cell lines (n = 3) compared to H3.3K27 WT (*n* = 4). * *p* < 0.05 compared to H3.3K27 WT group, independent-sample t test, two-tailed. un = unmodified, me1 = mono-methylation, me2 = di-methylation, me3 = tri-methylation, ac = acetylation

Using MRM, we determined histone N-terminal tail signatures (histone codes) in H3.3K27M mutant and wild type (WT) glioma cell lines and astrocytes (Supplementary material 2, Table 1). Histone codes were distinct by H3 mutation status (Fig. 1a). K27 di- and tri-methylation (K27me2/3) were significantly decreased in H3.3K27M mutant cells on H3.1 and H.3.3 isoforms (Fig. 1a, Supplementary material 3, 4). A significant difference in H3.1K27me3 was also observed, with a mean relative abundance of 15.9% versus 1.4% in mutant versus WT lines (Fig. 1b). A similar trend was observed on H3.3, with H3.3K27me3 relative abundance of 5.6% in H3.3K27 WT cells and only 0.6% in mutants (Fig. 1c). We observed greater K36 di- and tri-methylation in mutants, coupled with less abundant H3.1K36me (Fig. 1d, e). H3K79me2 was less abundant in H3.3K27M mutant cells compared to WT (mean = 8.3 and 16.3%, respectively, Fig. 1f).

Histone acetylation also differed based on mutation status. Acetylation at H4K16 was lower in H3.3K27 WT cells compared to mutant (31.5 and 48.2%, respectively Fig. 1g). H3.1K27me2 and H3.1K36me2 were the most common PTMs associated with H3.3K27M mutant peptide on multivariate analysis (*F* = 428.7, *p* < 0.01). Importantly, because the *H3F3A* mutation yielding the H3.3K27M mutant protein is heterozygous, we quantified relative abundance of H3.3K27 WT and H3.3K27M peptides in a given cell sample: 29.2% of H3.3K27 peptides in our mutant cell lines were H3.3K27M (SD = 6.6, range 22.2–35.4%, *n* = 3. Figure 1c). As expected, the H3.3K27M peptide was not detected in H3.3K27 WT cells.

### Glioma tissue histone codes are distinct by H3.3K27M mutation status

**Fig. 2:**
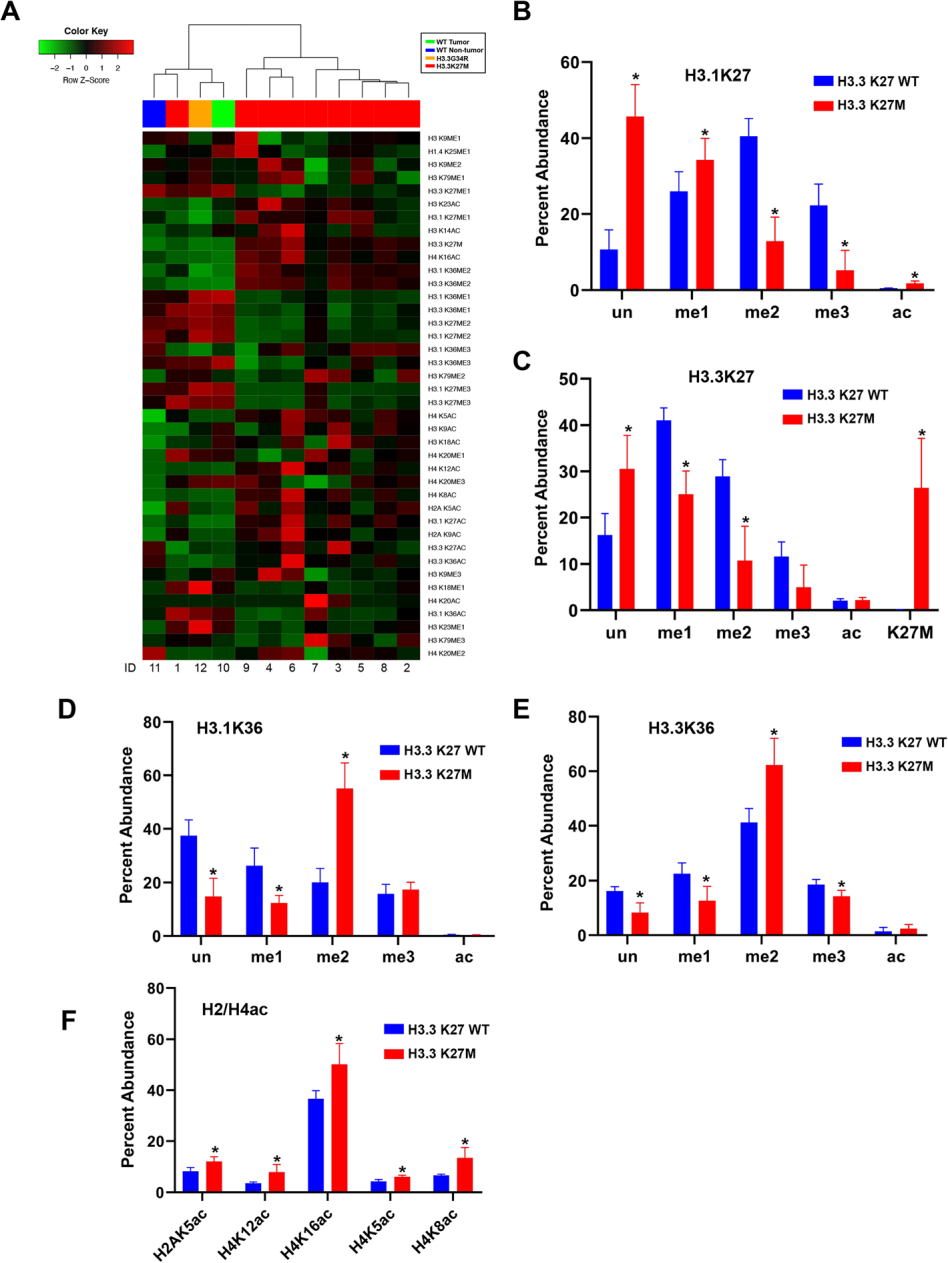
Tissue histone peptide modification states differ by H3.3K27M mutation status. **a** Unsupervised hierarchical clustering of glioma and normal brain tissue histone tail modification profiles reveals clustering by H3.3K27M mutation status. **b-e** Bar graph showing relative modifications at H3.1K27/K36 and H3.3K27/K36 in tissue specimens are distinct by H3.3K27M mutation status. **f** Statistically significant modifications of H2 and H4 acetylation are observed in H3.3K27M mutant tissues (n = 9) compared to H3.3K27 WT (*n* = 3).* *p* < 0.05 compared to H3.3K27 WT group, independent-sample t test, two-tailed. un = unmodification, me1 = mono-methylation, me2 = di-methylation, me3 = tri-methylation, ac = acetylation

Tissue histone PTM profiles also clustered by H3.3K27M mutation status (Fig. 2a). The relative abundance of 16 PTMs were statistically significantly different between H3.3K27M mutant and WT tissues (Supplementary material 5). As with cell lines, the greatest differences in PTMs between H3.3K27M mutant and WT tissues were K27 and K36 methylation states (Fig. 2b-e, Supplementary material 5). We also detected significantly greater acetylation of H2AK5, H4K5, H4K8, H4K12, and H4K16 (Fig. 2f). H3.3K27me1 and H4K16ac were the most common PTM states associated with H3.3K27M peptide abundance on multivariate analysis (*F* = 214.0, *p* < 0.01).

**Fig. 3:**
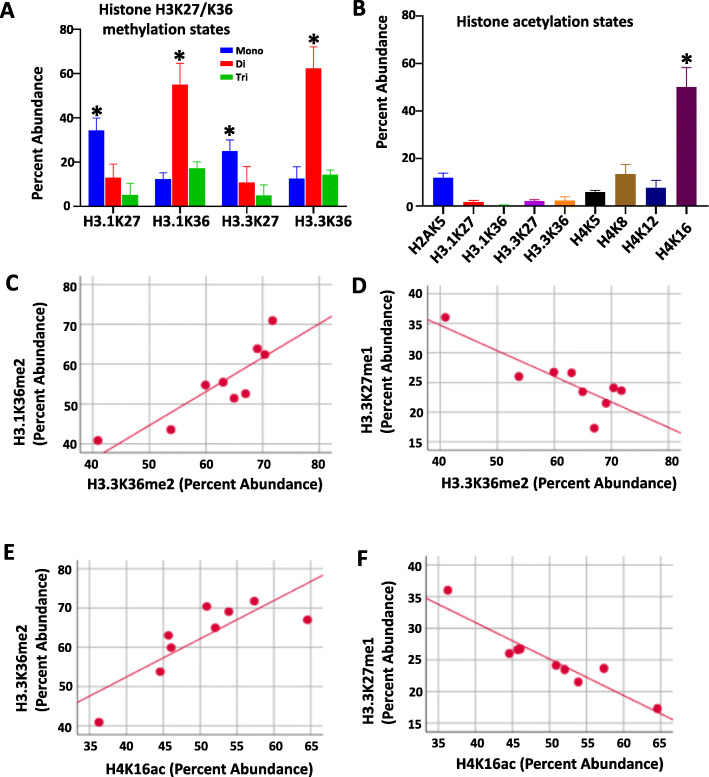
Predominant histone modification states in DIPG tumor tissue. **a** K27 mono-methylation and K36 di-methylation are predominant modification states on H3.1 and H3.3 tails in DIPG tumor tissue (*n* = 9). * *p* < 0.05 compared to all the other modification states (Tukey HSD, Post Hoc Tests, one way ANOVA). **b** H4K16ac is the predominant acetylated residue in DIPG tumor tissue (n = 9). * *p* < 0.05 compared to all the other modification states (Tukey HSD, Post Hoc Tests, one way ANOVA). **c**,**d** H3.3K36me2 abundance positively correlates with H3.1K36me2, and negatively correlates with H3.3K27me1 (Pearson Correlation = 0.87 and − 0.84, respectively, all *p* < 0.01. Pearson Correlation, two-tailed). **e**, **f** H4K16ac abundance positively correlates with H3.3K36me2, and negatively correlates with H3.3K27me1 (Pearson Correlation = 0.82 and − 0.94, respectively, all *p* < 0.01. Pearson Correlation, two-tailed)

As the greatest difference in relative abundance of PTM states in H3.3K27M mutant specimens were observed at H3K27, H3K36 and multiple H4 tail amino acids, we queried for the predominant modification states at these residues to determine correlations between specific modification states at these locations (Fig. 3). Overall, K27me1 and K36me2 were the predominant modifications observed on H3.1 and H3.3 isoforms (Fig. 3a), while acetylation was greatest at H4K16 (Fig. 3b). H3.3K36me2 abundance positively correlated with H3.1K36me2 levels, and negatively correlated with H3.3K27me1 (Fig. 3c, d). Also, H4K16ac abundance correlated positively with H3.3K36me2, and negatively with H3.3K27me1 (Fig. 3e, f).

We also quantified the relative abundance of H3.3K27 WT and H3.3K27M peptides in a given tissue sample. Similar to cell lines, 26.44% of H3.3K27 peptides in our mutant tumor specimens were H3.3K27M (SD = 10.7, range 5.2–44.5%, *n* = 9), with no mutant peptides detected in WT tissues.

Lastly, we compared DIPG cell and tumor tissue epiproteomic profiles. Overall, modifications on the H3.1/H3.3K27 N-terminal tail were similar in DIPG cell lines and tumor tissues. Specifically, there was no significant difference in abundance of the majority of H3.1/H3.3K27 peptides quantified, with differential fold change (FC) in abundance > 2 or < 0.5 for only six peptide modification states between DIPG cells and tissues (Table 2, Supplementary material 6). Significant differences in peptide modification states between groups were observed on H1.4K25, H3.3K36, H3K79, H3K9, and H4K20 peptides. Importantly, no difference in relative H3.3K27M peptide abundance was detected between DIPG tissue and cell lines (mean = 26.4 and 29.2, respectively, *p* = 0.686). Of note, unmodified peptides and modifications with < 5% relative abundance were excluded from this comparative analysis to ensure biological significance.

**Table 2:** Comparison of DIPG Tumor tissue and cell line epiproteomic profiles. Differential fold change (FC) in abundance (> 2 or < 0.5) between DIPG tumor tissue and cell lines was detected only in the six H3.1/H3.3K27 peptide modification states listed

Histone code	Mean		Fold_Change	p value
	Tissue	Cells	Tissue/Cells	
H3.3K36ac	2.4	15.8	0.2	< 0.01
H3K79me2	18.0	8.3	2.2	< 0.05
H3.3K36me1	12.7	5.3	2.4	< 0.05
H3K9ac	5.6	2.0	2.8	< 0.01
H4K20me3	8.5	2.2	3.9	< 0.01
H1.4K25me1	29.7	5.1	5.8	< 0.01

### Co-occurring histone modification states in tissue specimens

**Fig. 4:**
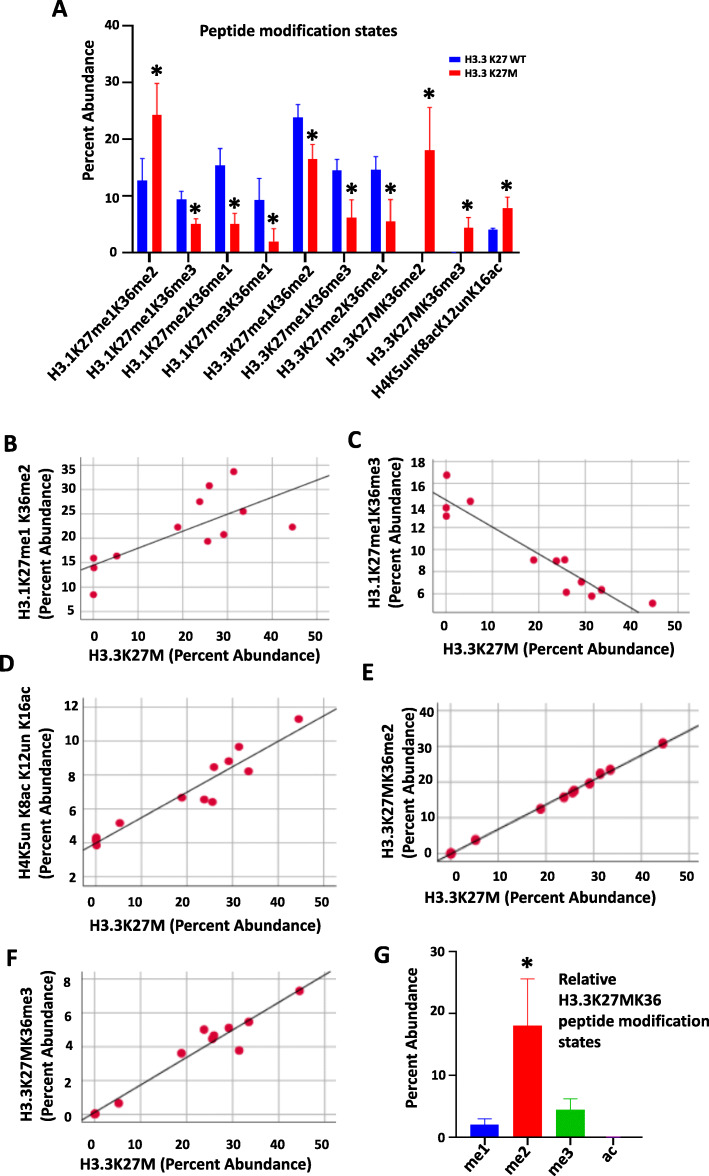
Distinct peptide modification states detected in DIPG tumor tissue. **a** Distinct combinations of modification states are observed on ten peptides in H3.3K27M mutant tumor tissue (n = 9) relative to H3.3K27 wild-type (WT) tissue (n = 3).* *p* < 0.05 compared to H3.3K27 WT group, Independent-sample t test, two-tailed. **b-f** Scatter plots showing the correlation between relative abundance of H3.1K27me1K36me2/me3, H4K5un K8acK12unK16ac, and H3.3K27MK36me2/3 peptides with the H3.3K27M peptide in DIPG tumor tissue (Pearson Correlation = 0.72, −0.79, 0.95, 0.99, and 0.97, respectively, all *p* < 0.01. Pearson Correlation, two-tailed, n = 12). **g** Relative abundance of H3.3K27MK36 peptide modification states in DIPG tumor tissue reveals K36me2 as the most abundant co-occurring peptide with H3.3K27M mutation peptide. * *p* < 0.05 compared to all the other groups (Tukey HSD, Post Hoc Tests, one way ANOVA, n = 9)

Because our method requires protein digestion for subsequent peptide mass spectrometry, determination of co-occurring PTMs at distant amino acid residues along the N-terminal tail is not possible. However, PTMs at proximal amino acids on the same peptide can be assessed. Therefore, to further explore the relationship between the H3.3K27M mutant peptide and histone PTM states, we investigated the PTMs of H3K27/K36 and H4K5/K8/K12/K16 peptides. The relative abundance of ten combinations of PTM states along these peptides were significantly different by tissue H3.3K27M mutation status (Fig. 4a, Supplementary material 7). Of these, H3.1K27me1K36me2, H3.3K27MK36me2, and H4K5unK8acK12unK16ac were increased in mutants compared to WT. These findings are consistent with the single amino acid analysis presented above. Further, we observed a positive correlation between H3.1K27me1K36me2 and H3.3K27M peptide abundance, and a negative correlation between H3.1K27me1K36me3 and H3.3K27M peptide abundance (Fig. 4b, c, Supplementary material 7). The abundance of the H4K5unK8acK12unK16ac peptide also positively correlated with H3.3K27M peptide abundance (Fig. 4d, Supplementary material 7). In contrast, H3.1K27me2K36me1, H3.1K27me3K36me1, H3.3K27me1K36me2/3 and H3.3K27me2K36me1 peptide abundance negatively correlated with H3.3K27M abundance (Supplementary material 7). On multivariate analysis, K36me2/3 were the most common modification states on the mutant H3.3K27MK36 peptide (*F* = 18,444.8, *p* < 0.01). As such, H3.3K27MK36me2 and H3.3K27MK36me3 peptide abundance also positively correlated with H3.3K27M abundance (Fig. 4e, f, Supplementary material 7). Importantly, di-methylation was the predominant modification state of the K36 residue on the H3.3K27MK36 peptide, while K36Ac was least abundant (Fig. 4g).

### Radiation treatment alters DIPG histone codes in vitro

**Fig. 5:**
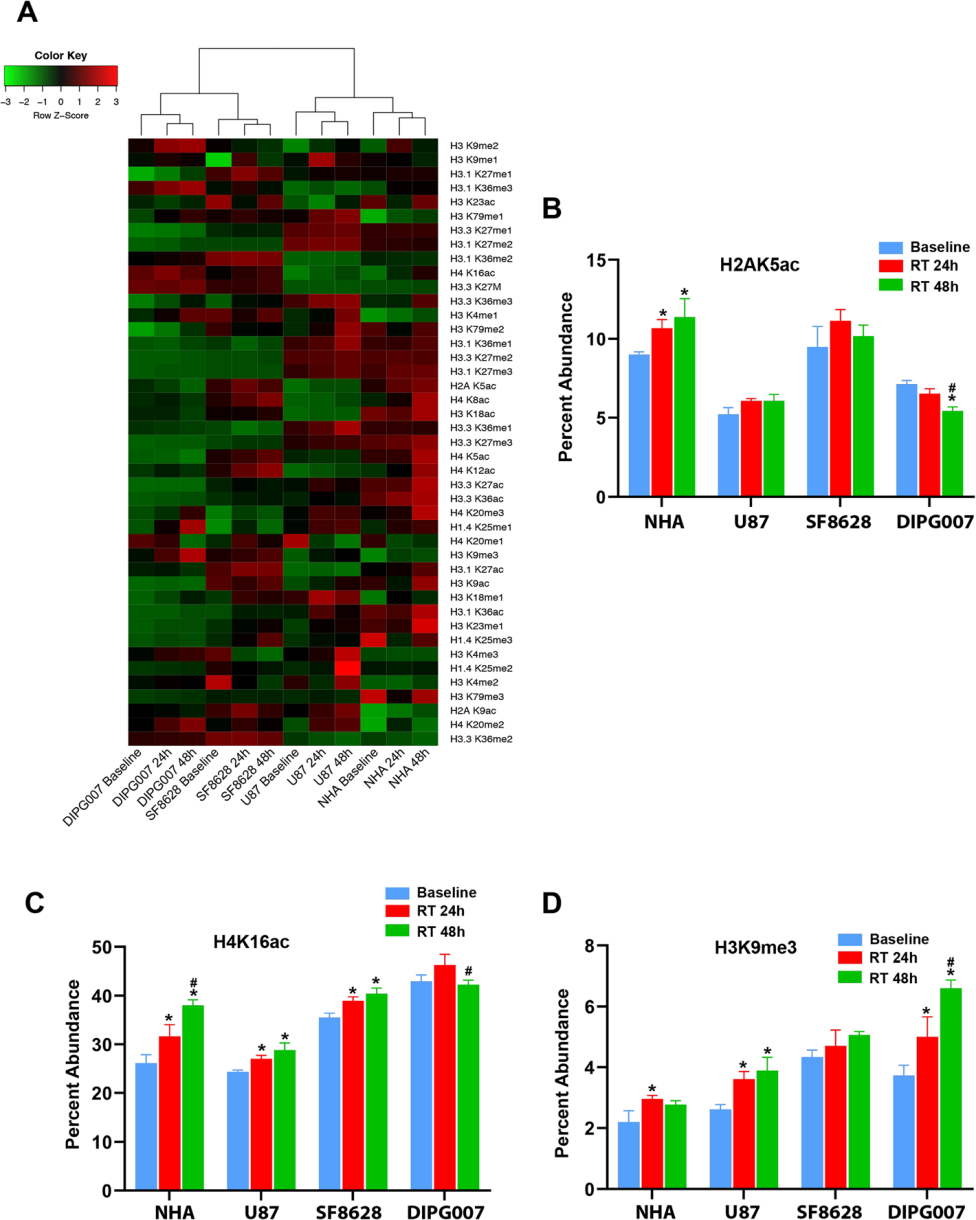
Radiation treatment alters relative histone peptide modification states in DIPG, in vitro. **a** Unsupervised hierarchical clustering of pediatric glioma cell line histone tail modification profiles reveals clustering by cell line and treatment condition. **b-d** Relative abundance of H2AK5ac, H4K16ac and H3K9me3 peptides are distinct after radiation treatment. * *p* < 0.05 compared to Baseline; # *p* < 0.05 compared to 24 h post-RT treatment (Tukey HSD, Post Hoc Tests, one way ANOVA)

As radiation treatment (RT) is the standard therapy for DIPG, we examined the effects of RT on the glioma histone code in vitro. We treated H3.3K27M mutant DIPG cells (SF8628, DIPG007), H3.3K27 WT glioma cells (U87) and normal astrocytes (NHA) with 9 Gy radiation, and performed targeted histone mass spectrometry at 24 and 48 h post-treatment. Cell histone codes clustered first by origin, then by treatment condition (Fig. 5a). Comparative analysis revealed distinct differences in relative peptide abundance after RT, most notably in acetylated peptides (Supplementary material 8). In DIPG007 cells, the relative abundance of H2AK5ac decreased with RT at 48 h (Fig. 5b). In contrast, H2AK5ac abundance increased in NHA at 24 and 48 h, with no significant change in SF8628 or U87 (Fig. 5b). At 24 and 48 h post-RT, all lines except DIPG007 showed a significant increase in H4K16ac (Fig. 5c, Supplementary material 7). H3K9me3 also significantly increased in NHA, U87, and DIPG007, and trended towards a significant increase in SF8628, after RT (Fig. 5d, Supplementary material 8).

To determine the potential biological impact of observed differences in histone modifications after RT relative to controls, we compared cell gene expression profiles in these same treatment groups. On functional pathways analysis of genes identified as statistically significantly differentially expressed by RT treated cells relative to controls (fold change in expression > 2 or < − 2, adjusted *p*-value < 0.05), we identified DNA double-strand break repair as a top canonical pathway activated in RT treated DIPG cell lines (p-value 1.10 × 10^− 21^, Supplementary material 9). The top implicated molecular and cellular function in RT treated cells, as well as the top network of molecular interaction, was DNA replication, recombination and repair (p-value 2.06 × 10^− 6^),

### Bromodomain inhibition alters DIPG histone codes in vitro

**Fig. 6:**
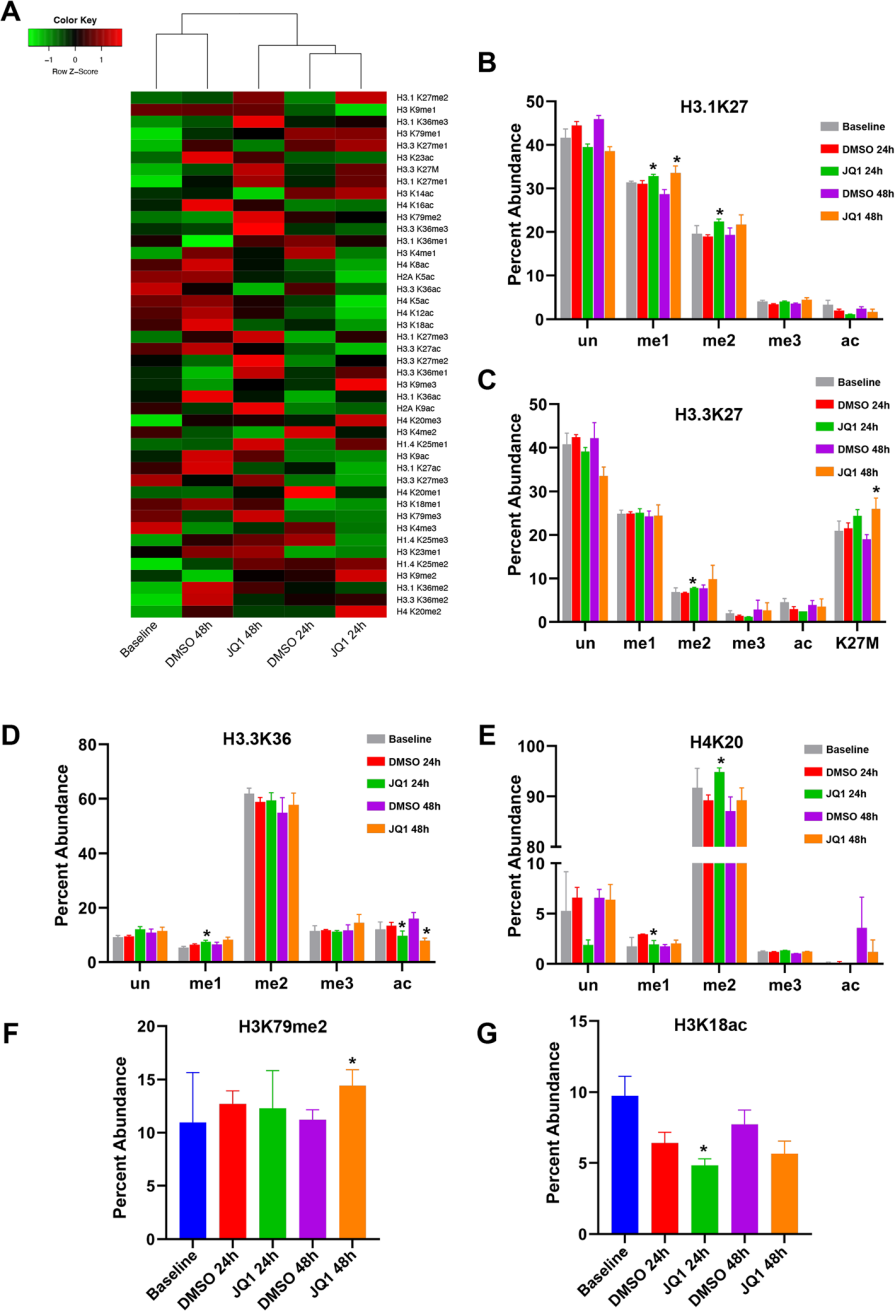
Bromodomain inhibition with JQ1 alters relative histone peptide modification states in DIPG, in vitro. **a** Unsupervised hierarchical clustering of DIPG cell line SF8628 histone tail modification profiles reveals clustering by treatment condition. **b-e** Relative abundance of H3.1/3.3 K27, H3.3K36, and H4K20 peptide modification states in SF8628 cells are distinct after JQ1 treatment. **f-g** Relative abundance of H3K79me2 and H3K18ac peptides in SF8628 cell lines are distinct after JQ1 treatment. * *p* < 0.05 compared to DMSO group (independent-sample t test, two-tailed)

We previously reported pre-clinical efficacy of JQ1, a BET/Bromodomain inhibitor (BRDi), in DIPG [7]. We observed that BRD proteins are enriched at acetylated residues on histone H3 in DIPG cells and animal models, and that JQ1 treatment results in decreased global H3K27ac, with restoration of global H3K27 methylation [7]. To further elucidate the epigenetic effects of JQ1 in DIPG, we examined its effects on histone codes in SF8628, DIPG007, NHA, and U87 cells. As observed with RT, PTM profiles clustered by cell line, then treatment condition (Supplementary material 10). When analyzed individually, cell profiles cluster by treatment condition (Fig. 6a, Supplementary material 11). In DIPG007 cells, H3.3K27me1 decreased by 1.5% at 48 h after JQ1 treatment compared to vehicle control, representing the only significant change in modification state observed at that time point (Supplementary material 11). A small increase in H3.3K27M abundance was also detected in DIPG007 at 48 h (31.4% vs 26.9%, *p* = 0.067, Supplementary material 11). Surprisingly, in DIPG007, H3.3K27ac abundance was not significantly different from control at 48 h post-treatment, though a marginal decrease was observed at 24 h (1.65 and 1.95%, respectively, *p* = 0.065, Supplementary material 11). These results were validated in DIPG007 and SF8628 cells via western blot for H3K27M and H3K27ac (Supplementary material 12).

In contrast, we observed statistically significant differences in abundance of multiple PTMs in SF8628 cells in response to JQ1 (Supplementary material 11). Increased H3.1K27me1 and decreased H3.3K36ac were seen at both 24 h and 48 h (Fig. 6b, Table 3, Supplementary material 11). Additional changes in relative modification states were observed after either 24 or 48 h post-treatment. For example, increased abundance of H3.1/H3.3K27me2, H3.3K36me1, and H4K20me2 were seen 24 h (Fig. 6b-e, Table 3) and H3K79me2 at 48 h (Fig. 6f, Table 3, Supplementary material 11). In addition, acetylation of H3K18 significantly decreased at 24 h (Fig. 6g, Table 3, Supplementary material 11). Surprisingly, we also observed a significant increase in H3.3K27M peptides at 24 and 48 h after JQ1 treatment in SF8628 cells (Fig. 6b, Table 3, Supplementary material 11).

**Table 3:** Effects of bromodomain inhibition with JQ1 on relative histone peptide abundance in SF8628 cells

Histone code	Mean					Fold (JQ1/DMSO)		***p***_SF8628	
	Baseline	DMSO 24 h	DMSO 48 h	JQ1 24 h	JQ1 48 h	24 h	48 h	24 h	48 h
**H3.1K27me1**	**31.4**	**31.1**	**28.7**	**32.8**	**33.5**	**1.1**	**1.2**	**< 0.05**	**< 0.05**
**H3.1K27me2**	**19.6**	**19.0**	**19.3**	**22.4**	**21.7**	**1.2**	**1.1**	**< 0.01**	**0.21**
**H3.3K27me2**	**6.9**	**6.7**	**7.7**	**7.8**	**9.8**	**1.2**	**1.3**	**< 0.01**	**0.33**
**H3.3K27M**	**21.0**	**21.5**	**19.0**	**24.4**	**26.0**	**1.1**	**1.4**	**< 0.05**	**< 0.05**
**H3.3K36me1**	**5.4**	**6.4**	**6.5**	**7.5**	**8.3**	**1.2**	**1.3**	**< 0.05**	**0.07**
**H3.3K36ac**	**12.0**	**13.5**	**16.0**	**9.7**	**7.9**	**0.7**	**0.5**	**< 0.05**	**< 0.01**
**H3K18ac**	**9.7**	**6.4**	**7.7**	**4.8**	**5.7**	**0.8**	**0.7**	**< 0.05**	**0.05**
**H3K79me2**	**11.0**	**12.7**	**11.2**	**12.3**	**14.4**	**1.0**	**1.3**	**0.86**	**< 0.05**
**H4K20me1**	**1.7**	**2.9**	**1.7**	**1.9**	**2.0**	**0.7**	**1.2**	**< 0.05**	**0.29**
**H4K20me2**	**91.7**	**89.2**	**87.1**	**94.8**	**89.2**	**1.1**	**1.0**	**< 0.01**	**0.38**

Changes in histone PTM profiles were also observed in H3.3K27 WT cell lines after exposure to JQ1. The relative abundance of multiple PTMs were observed in U87 cells, including H3.1K27me2/me3, H3.1K36me1/me3, H3.3K36me1/ac, H3K14ac, H3K79me1, H3K4me1, and H4K5ac (Table 4, Supplementary material 11). Lastly, distinct changes were observed in NHAs after JQ1 treatment, including methylation of H3.1K27, H3.1/3.3 K36, and H3K79me1, and acetylation of H2AK5, H3.3K36, H3K18, and H4K16ac (Table 5, Supplementary material 11).

**Table 4:** Effects of bromodomain inhibition with JQ1 on relative histone peptide abundance in U87 cells

Histone code	Mean					Fold (JQ1/DMSO)		***p***_U87	
	Baseline	DMSO 24 h	DMSO 48 h	JQ1 24 h	JQ1 48 h	24 h	48 h	24 h	48 h
**H3.1K27me2**	**34.7**	**34.2**	**35.2**	**37.0**	**38.6**	**1.1**	**1.1**	**< 0.05**	**< 0.05**
**H3.1K27me3**	**10.0**	**11.0**	**12.2**	**13.1**	**14.4**	**1.2**	**1.2**	**< 0.05**	**< 0.05**
**H3.1K36me1**	**19.9**	**19.6**	**18.3**	**17.9**	**19.0**	**0.9**	**1.0**	**< 0.05**	**0.39**
**H3.1K36me3**	**15.4**	**16.4**	**15.3**	**14.9**	**14.1**	**0.9**	**0.9**	**< 0.05**	**< 0.05**
**H3.3K36me1**	**10.7**	**11.1**	**10.4**	**11.4**	**12.5**	**1.0**	**1.2**	**0.51**	**< 0.05**
**H3.3K36ac**	**13.3**	**11.9**	**14.1**	**10.9**	**9.6**	**0.9**	**0.7**	**0.30**	**< 0.01**
**H3K14ac**	**45.0**	**37.3**	**28.5**	**30.2**	**26.8**	**0.8**	**0.9**	**< 0.05**	**0.38**
**H3K79me1**	**24.8**	**27.7**	**26.6**	**23.9**	**23.7**	**0.9**	**0.9**	**< 0.01**	**0.14**
**H3K4me1**	**21.1**	**17.6**	**16.7**	**12.8**	**15.5**	**0.7**	**0.9**	**< 0.01**	**0.69**
**H4K5ac**	**4.9**	**5.6**	**5.4**	**4.6**	**4.6**	**0.8**	**0.8**	**< 0.01**	**< 0.05**

**Table 5:** Effects of bromodomain inhibition with JQ1 on relative histone peptide abundance in NHA cells

Histone code	Mean					Fold (JQ1/DMSO)		***p***_NHA	
	Baseline	DMSO 24 h	DMSO 48 h	JQ1 24 h	JQ1 48 h	24 h	48 h	24 h	48 h
**H2AK5ac**	**8.8**	**6.4**	**6.2**	**3.9**	**4.7**	**0.6**	**0.8**	**0.06**	**< 0.05**
**H3.1K27me3**	**19.7**	**18.3**	**18.2**	**22.0**	**24.0**	**1.2**	**1.3**	**< 0.05**	**< 0.05**
**H3.1K36me1**	**14.6**	**14.5**	**13.9**	**16.4**	**15.9**	**1.1**	**1.1**	**0.21**	**< 0.05**
**H3.1K36me2**	**27.6**	**29.2**	**29.7**	**25.6**	**24.1**	**0.9**	**0.8**	**0.08**	**< 0.05**
**H3.3K36me1**	**8.7**	**9.2**	**8.5**	**10.6**	**11.0**	**1.2**	**1.3**	**0.17**	**< 0.01**
**H3.3K36ac**	**8.5**	**8.8**	**10.0**	**6.3**	**7.1**	**0.7**	**0.7**	**< 0.05**	**< 0.01**
**H3K18ac**	**10.1**	**8.1**	**7.3**	**6.2**	**6.0**	**0.8**	**0.8**	**0.10**	**< 0.05**
**H3K79me1**	**14.3**	**15.2**	**16.1**	**17.5**	**14.2**	**1.2**	**0.9**	**0.27**	**< 0.01**
**H4K16ac**	**28.5**	**27.5**	**27.3**	**26.2**	**30.8**	**1.0**	**1.1**	**0.66**	**< 0.01**

To determine the potential biological impact of observed differences in histone modifications after BRD inhibition relative to controls, we also compared cell gene expression profiles in these treatment groups. On functional pathways analysis of genes identified as statistically significantly differentially expressed by JQ1 treated cells relative to controls (fold change in expression > 2 or < − 2, adjusted *p*-value < 0.05), we identified nucleotide excision repair as the top canonical pathway activated in JQ1 treated DIPG cell lines (p-value 2.51 × 10^− 48^, Supplementary material 9). Other canonical pathways implicated by JQ1 treated cell gene expression profiles included DNA mismatch repair via non-homologous end joining (NEMJ, p-value 2.53 × 10^− 32^), while G2/M DNA damage check point regulation was the top toxicology list match (p-value 2.68 × 10^− 6^). Top upstream regulator effect networks implicated ANXA7 activation (resulting in DNA binding and conformational modification, score 9.615), and TP73 inhibition (resulting in apoptosis, score − 15.254) in JQ1 treated DIPG cells relative to controls.

## Discussion

DIPG is an incurable pediatric brain tumor, with a high rate of somatic missense mutations in genes encoding histone H3.1 and H3.3 proteins. Here, we present comprehensive characterization of histone N-terminal tail modification states in pediatric glioma specimens, including H3.3K27M mutant DIPG tissue, using novel, high-throughput proteomic analysis, revealing the effects of radiotherapy and BRDi on the tumor epigenetic landscape. The greatest differences in PTMs between H3K27M mutant and WT specimens were observed at H3K27, H3K36, and H4K16ac, while H3K36me2 was the highest co-occurring modification on the H3.3K27M peptide, and H4K16 was the most acetylated residue. These results suggest that H3K36me2 and H4K16ac may serve as epigenetic signatures in DIPG, and that targeting these modification states may represent a potential therapeutic strategy.

Multiple groups have identified distinct DIPG molecular subtypes, with H3.3K27M mutant tumors associated with shorter survival [22–30]. We found that H3.3K27M significantly affects the histone PTM landscape, with cell and tissue profiles clustering by mutation status. Further, a decrease in global H3K27me2/3 has been observed in H3.3K27M tumors, and our results corroborate these data, [5, 7, 8, 10, 12, 13, 31]. Of note, a new subgroup of DIPG lacking H3K27M mutation but still exhibiting genomic H3K27me3 loss was recently reported [32]: we did not observe this phenomenon in our cohort (Supplementary material 4). Our H3K27M mutant specimens also exhibit distinct modification states at H3K36 and H4K16. Previous work showed that PRC2 loss coincides with increased H3K36me2, and that an increase in H3K36 methylation impacts methylation at H3K27 [33–35]. Our results also confirm an increase in H3K36me2 in DIPG specimens on the H3.3K27M mutant peptide, as previously detected via mass spectrometry of H3.3K27M transgenic PDGF-driven murine glioblastoma [13]. Further, we detected high H4K16ac abundance in DIPG cells and tissue specimens. A recently published report characterizing high-throughput screening with nucleosome substrate methods demonstrates small-molecule inhibitors of the human histone lysine methyltransferase NSD2 [36], the writer of mono- and di-methylation of H3K36 [37]; another report identified DC_M01_7 as a novel inhibitor of MOF, the H4K16ac-specific acetyltransferase [38, 39]. Therefore, H3K36me2 and H4K16ac may represent potential therapeutic targets in DIPG, and further investigation of this strategy is warranted.

Using our method, we also characterized H3K27 and K36 modification states *in cis*, confirming me2/3 as the predominant modifications, and acetylation as the least abundant, on the H3.3K27MK36 peptide. We observed positive correlation between H3.3K27M and H3.1K27K36me2, and negative correlation between H3.3K27M and H3.1K27K36me3. Taken together, these results confirm that H3.3K27M affects PTMs on both mutant and WT H3.1 and H3.3 N-terminal tails. Importantly, our study is the first, to our best knowledge, to quantify the relative amount of mutant H3.3K27M peptide in DIPG tumor specimens. It is reported that relative abundance of mutant epidermal growth factor receptor (EGFR) can predict benefit from EGFR-tyrosine kinase inhibitors for advanced non-small-cell lung cancer [40]. Longitudinal quantification of the H3.3K27M peptide in patient-derived specimens might similarly serve as a predictive biomarker for targeted therapy, and hence is worthy of consideration when designing future clinical trials.

Radiation therapy (RT) is standard treatment for DIPG, providing transient symptom relief but no change in disease outcome [41]. Unfortunately, the mechanism of radiation resistance is unknown, and radio-sensitizers provide no benefit [42]. Patients with the H3.3K27M mutation also respond more poorly to RT [24]. Our study reveals RT can induce cell-specific and peptide-specific changes in histone acetylation, underscoring the heterogeneity of DIPG and warranting consideration when using histone deacetylase / acetyltransferase inhibitors in combination with RT. Importantly, H3K9me3 abundance increased in all four cell lines after RT, which kills rapidly proliferating cancer cells by inducing DNA damage beyond cellular capacity for repair, including DNA double-strand breaks (DSBs) [43–45]. DSB repair is linked to rapid changes in epigenetic modifications, including increased H3K9 methylation, to recruit DNA repair proteins. Importantly, H3K9 methyltransferase G9a inhibition (BIX-01294) increases radio-sensitivity in glioma [46]. Indeed, on functional pathways analysis of cell gene expression profiles, we found DSB repair pathways implicated in RT treated cell lines relative to controls. These data suggest that combination therapy RT with G9a inhibition, aimed at reducing H3K9me3 levels and hence DNA DSB repair, may also be a useful therapeutic strategy in DIPG.

We found distinct DIPG histone codes after treatment with JQ1, a BET/ bromodomain inhibitor. BET domain proteins are epigenetic readers that bind acetylated histones via their bromodomain region to regulate gene transcription [46–48]. BET inhibitors dislodge acetylated histone readers from chromatin, leading to oncogene repression [49–51]. JQ1 is the best known BET inhibitor, and induces a dose-dependent reduction in DIPG cell viability and genomic H3K27ac in vitro and in vivo [7, 52, 53]. Our results showed increased mono- and/or di-methylation of multiple residues with JQ1 treatment, consistent with previous reports that JQ1 globally reduces acetylation and increases methylation [7]. Since mono-methylation of H3K27, H3K79, and H4K20 are all linked to gene activation, whereas tri-methylation of H3K27 and H3K79 are linked to repression [54], these findings may provide additional insight into the mechanism by which BRDi alters tumorigenic gene expression in DIPG. H4K20me, which is functional in DNA repair, represents a binding site for the 53BP1 protein; H3K9me3 and H4K20me3 represent epigenetic markers important to the function of 53BP1 in non-homologous end joining (NHEJ) repair [55]. In line with these known functions, pathways analysis of differential gene expression profiles implicated nucleotide excision repair and DNA mismatch repair via NHEJ as top activated pathways in JQ1 treated cells, compared to controls. As such, additional studies of the mechanism by which JQ1 may augment response to RT are necessary. Lastly, we also observed that reduction of histone acetylation in DIPG was greatest at 24 h, which may be important to consider when determining JQ1 dosing regimens.

Because fresh DIPG tissue is very rare, we chose to analyze archival post-mortem tissue specimens for this study, raising the possibility of treatment-induced effects. However, the patterns of peptide abundance observed in tumor tissues were similar to treatment-naïve cell lines. Analysis of treatment fresh tumor tissue specimens with this approach will be crucial to teasing out tumor biology from the effects of therapy. Optimization of our technical approach to minimize the amount of starting tumor tissue necessary for this purpose is currently underway.

## Conclusions

In summary, targeted histone tail mass spectrometry of high grade glioma cell lines and tissues, including rare DIPG tumor tissue specimens, reveals distinct peptide modification states by H3.3K27M mutation status. Our study is the first to quantify the H3.3K27M peptide in DIPG, representing an exciting new opportunity to apply this technique on clinical specimens at diagnosis, and potentially throughout the course of treatment to guide clinical management. We also distinct epigenetic signatures of DIPG, including H3K36me2 and H4K16ac, which may represent novel therapeutic vulnerabilities for more effective treatment of this devastating disease.

## Supplementary Information

**Additional file 1:** **S1.** Table of monitored peptide transitions.

**Additional file 2:** **S2.** Targeted mass spectrometry for histone tail analysis. Overview of study design and analysis of histone tail post-translational modification states in pediatric glioma specimens. Tumor tissue specimens were obtained post-mortem or during the course of treatment. Patient-derived tumor cell lines were established from tissue specimens. Extracted histones were analyzed using targeted mass spectrometry of the histone H3 and H4 N-terminal tail, with quantitation of histone post-translational modifications (me1, me2, me3, ac) and unmodified peptide on lysine (K) residues.

**Additional file 3:** **S3.** Table of results from targeted histone tail mass spectroscopy (Microsoft Excel format). Statistically significant differences in histone acetylation and methylation are observed in glioma cell lines by H3.3K27M mutation status (Independent-sample t test, two-tailed).

**Additional file 4:** **S4.** H3.3K27M, H3.1K27me3, and H3.3K27me3 peptide abundance in cell lines. Targeted mass spectroscopy reveals differences in H3.3K27M, H3.1K27me3, and H3.3K27me3 peptide abundance in pediatric glioma and adult glioma cells, and normal human astrocytes. Of note, loss of H3.1K27 and H3.3K27 trimethylation is observed in H3.3K27M cell lines.

**Additional file 5:** **S5.** H3.3K27M mutation is associated with distinct histone modification states (Microsoft Excel format). Statistically significant differences in histone acetylation and methylation are observed in glioma tissues by H3.3K27M mutant status (Independent-sample t test, two-tailed).

**Additional file 6:** **S6.** Modification states along short peptides (Microsoft Excel format). Distinct combinations of histone peptide modification states with H3.3K27M mutation status in tissues (Independent-sample t test, two-tailed).

**Additional file 7:** **S7.** Radiation treatment is associated with changes in histone modification states in vitro (Microsoft Excel format). Statistically significant differences in histone acetylation and methylation are observed in NHA, U87, SF8628, and DIPG007 cells treated with radiation (one-way ANOVA).

**Additional file 8:** **S8.** Bromodomain inhibition with JQ1 is associated with changes in histone modification states in vitro. Unsupervised analysis of histone tail modification profiles of glioma cell lines (SF8628, DIPG007 and U87) and normal astrocytes (NHA) reveals clustering by cell line and treatment condition.

**Additional file 9:** **S9.** Peptide modification states observed after bromodomain inhibition with in vitro. (Microsoft Excel format). Statistically significant differences in histone acetylation and methylation are observed in NHA, U87, SF8628, and DIPG007 cells after bromodomain inhibition with JQ1 (Independent-sample t test, two-tailed).

**Additional file 10:** **S10.** Functional pathways analysis of differentially expressed genes after RT and Bromodomain inhibition. A Differentially expressed genes by DIPG cells after 48 h RT compared to DMSO control enrich for DNA double-strand break repair as a top canonical pathway on functional analysis (*p*-value 1.10 × 10^− 21^). B Differentially expressed genes by DIPG cells after 48 h JQ1 treatment compared to DMSO control enrich for nucleotide excision repair as the top canonical pathway (p-value 2.51 × 10^− 48^).

**Additional file 11:** **S11.** Validation of changes in H3K27M and H3K27Ac in DIPG cells after Bromodomain inhibition. A Western blot of extracted histones for H3K27M and H3K27Ac from DIPG cell lines SF8628 and DIPG007 after 24 h of Bromodomain inhibition was performed in triplicate and compared to DMSO treated controls. B, C Bar graphs depicting mean OD value ± SEM across replicates for each treatment group, with *p*-values for between group comparisons (Independent-sample t test, two-tailed).

**Additional file 12.**

## Data Availability

All data generated or analyzed during this study are included in this article.

## Abbreviations

DIPG: Diffuse intrinsic pontine glioma
BRDi: Bromodomain inhibition
PTMs: Post-translational modifications
BRD2/4: Bromodomain proteins 2 and 4
BET: Bromodomain and extra terminal domain
WT: wild-type
K27me2/3: K27 di- and tri-methylation
RT: Radiation treatment
DSBs: DNA double-strand breaks
