# Supplementary material for: Histone tail analysis reveals H3K36me2 and H4K16ac as epigenetic signatures of diffuse intrinsic pontine glioma
Source: J Exp Clin Cancer Res. 2020 Nov 25;39:261. doi: 10.1186/s13046-020-01773-x (PMC7687710; doi:10.1186/s13046-020-01773-x)
Supplement: Supplementary file 2 — Additional file 2: S2. Targeted mass spectrometry for histone tail analysis. Overview of study design and analysis of histone tail post-translational modification states in pediatric glioma specimens. Tumor tissue specimens were obtained post-mortem or during the course of treatment. Patient-derived tumor cell lines were established from tissue specimens. Extracted histones were analyzed using targeted mass spectrometry of the histone H3 and H4 N-terminal tail, with quantitation of histone post-translational modifications (me1, me2, me3, ac) and unmodified peptide on lysine (K) residues. [file 13046_2020_1773_MOESM2_ESM.pptx]

## Slide 1
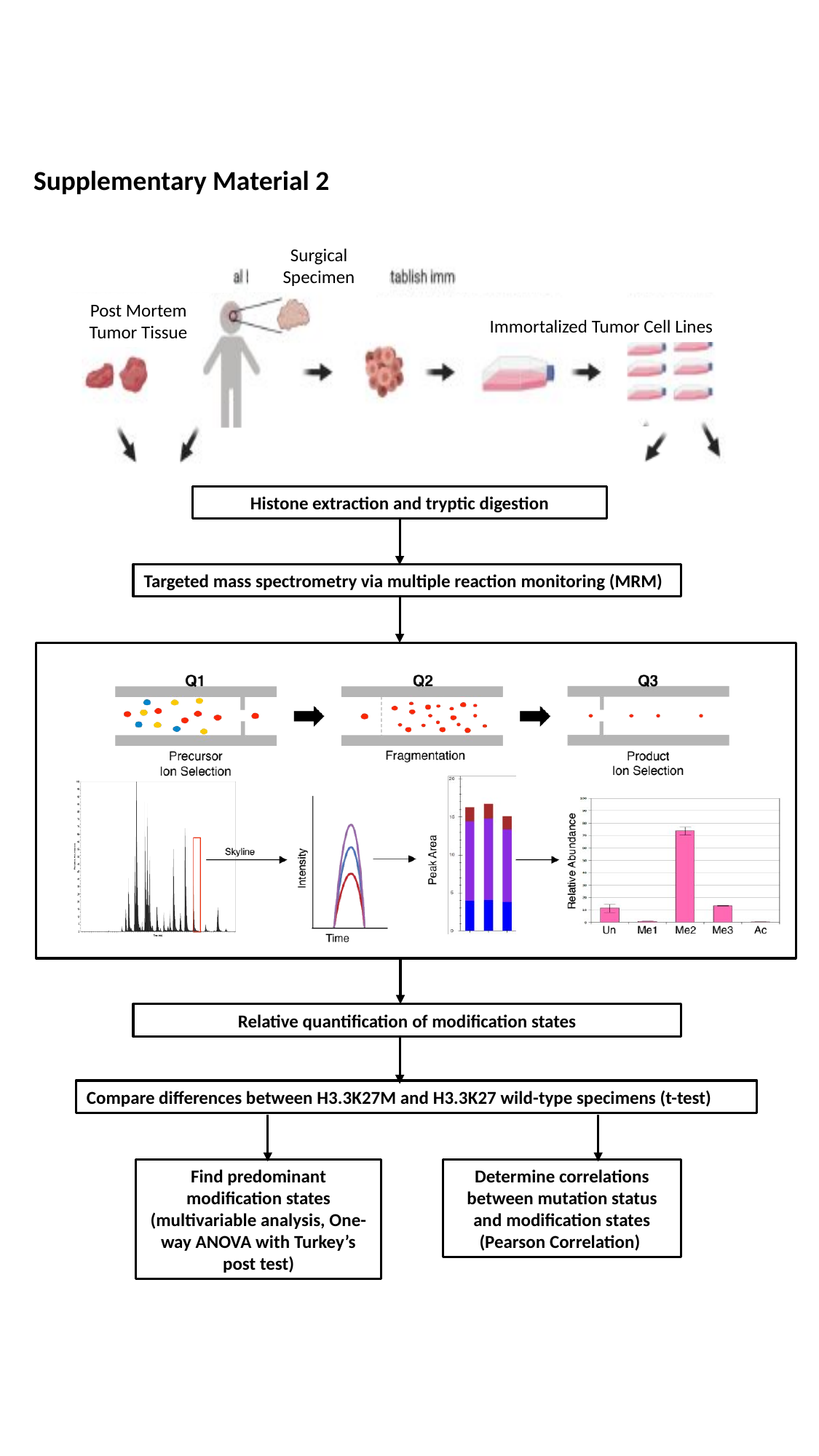

Supplementary Material 2
Surgical Specimen
Post Mortem Tumor Tissue
Immortalized Tumor Cell Lines
Histone extraction and tryptic digestion
Targeted mass spectrometry via multiple reaction monitoring (MRM)
Relative quantification of modification states
Compare differences between H3.3K27M and H3.3K27 wild-type specimens (t-test)
Find predominant modification states (multivariable analysis, One-way ANOVA with Turkey’s post test)
Determine correlations between mutation status and modification states (Pearson Correlation)
