# Supplementary figures and images for: Histone tail analysis reveals H3K36me2 and H4K16ac as epigenetic signatures of diffuse intrinsic pontine glioma
Source: J Exp Clin Cancer Res. 2020 Nov 25;39:261. doi: 10.1186/s13046-020-01773-x (PMC7687710; doi:10.1186/s13046-020-01773-x)

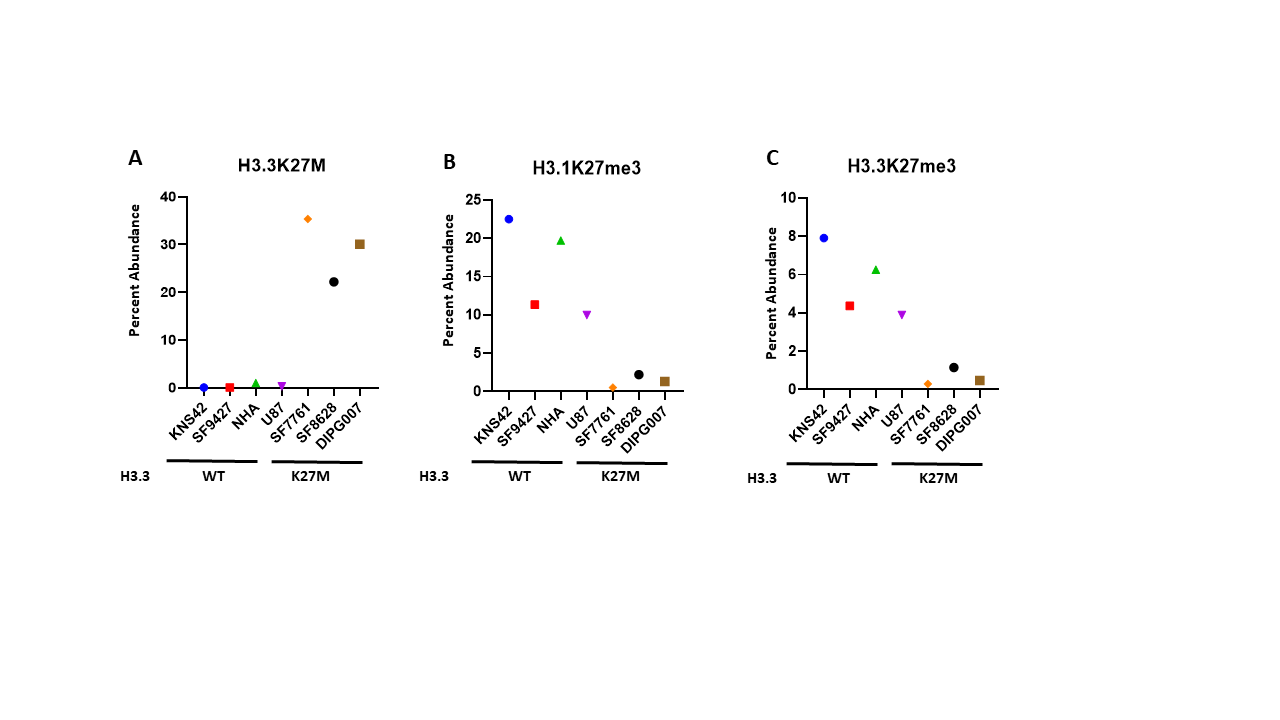

Supplement: Supplementary file 4 — Additional file 4: S4. H3.3K27M, H3.1K27me3, and H3.3K27me3 peptide abundance in cell lines. Targeted mass spectroscopy reveals differences in H3.3K27M, H3.1K27me3, and H3.3K27me3 peptide abundance in pediatric glioma and adult glioma cells, and normal human astrocytes. Of note, loss of H3.1K27 and H3.3K27 trimethylation is observed in H3.3K27M cell lines. [file 13046_2020_1773_MOESM4_ESM.tif]

## Slide 1
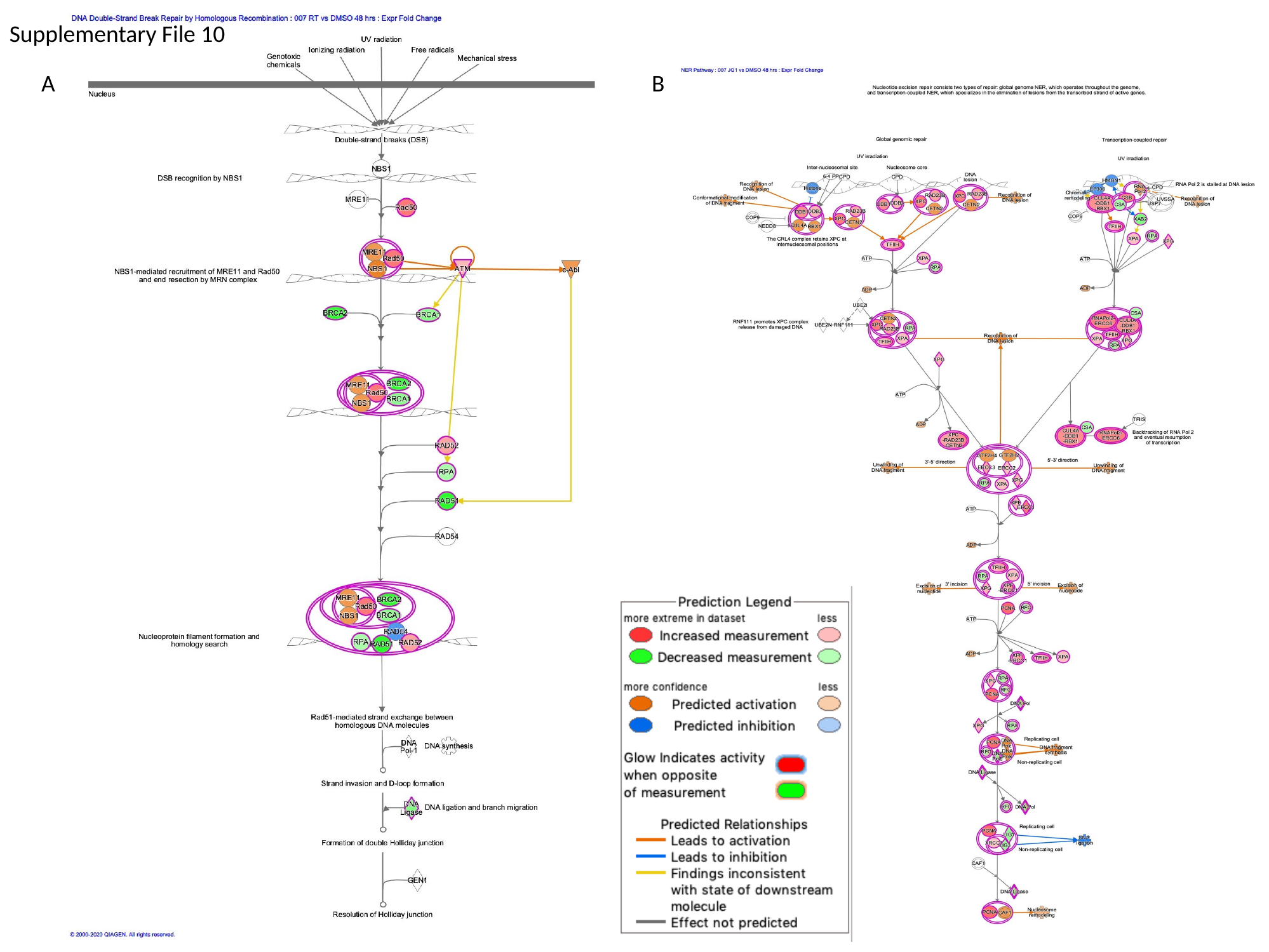

Supplementary File 10
A
B

Supplement: Supplementary file 9 — Additional file 9: S9. Peptide modification states observed after bromodomain inhibition with in vitro. (Microsoft Excel format). Statistically significant differences in histone acetylation and methylation are observed in NHA, U87, SF8628, and DIPG007 cells after bromodomain inhibition with JQ1 (Independent-sample t test, two-tailed). [file 13046_2020_1773_MOESM9_ESM.pptx]

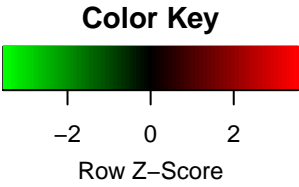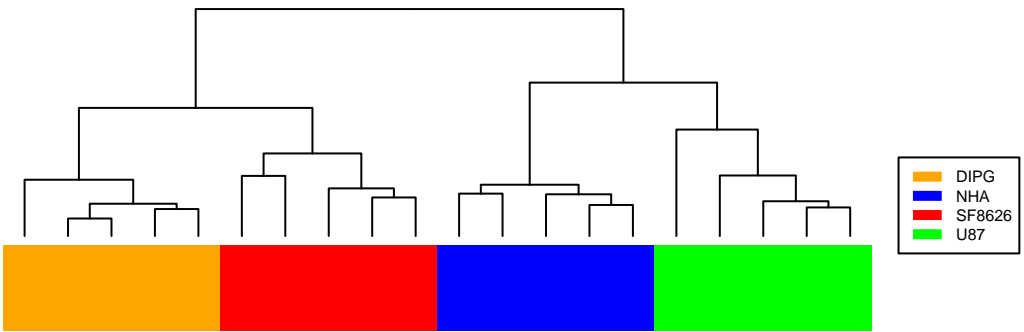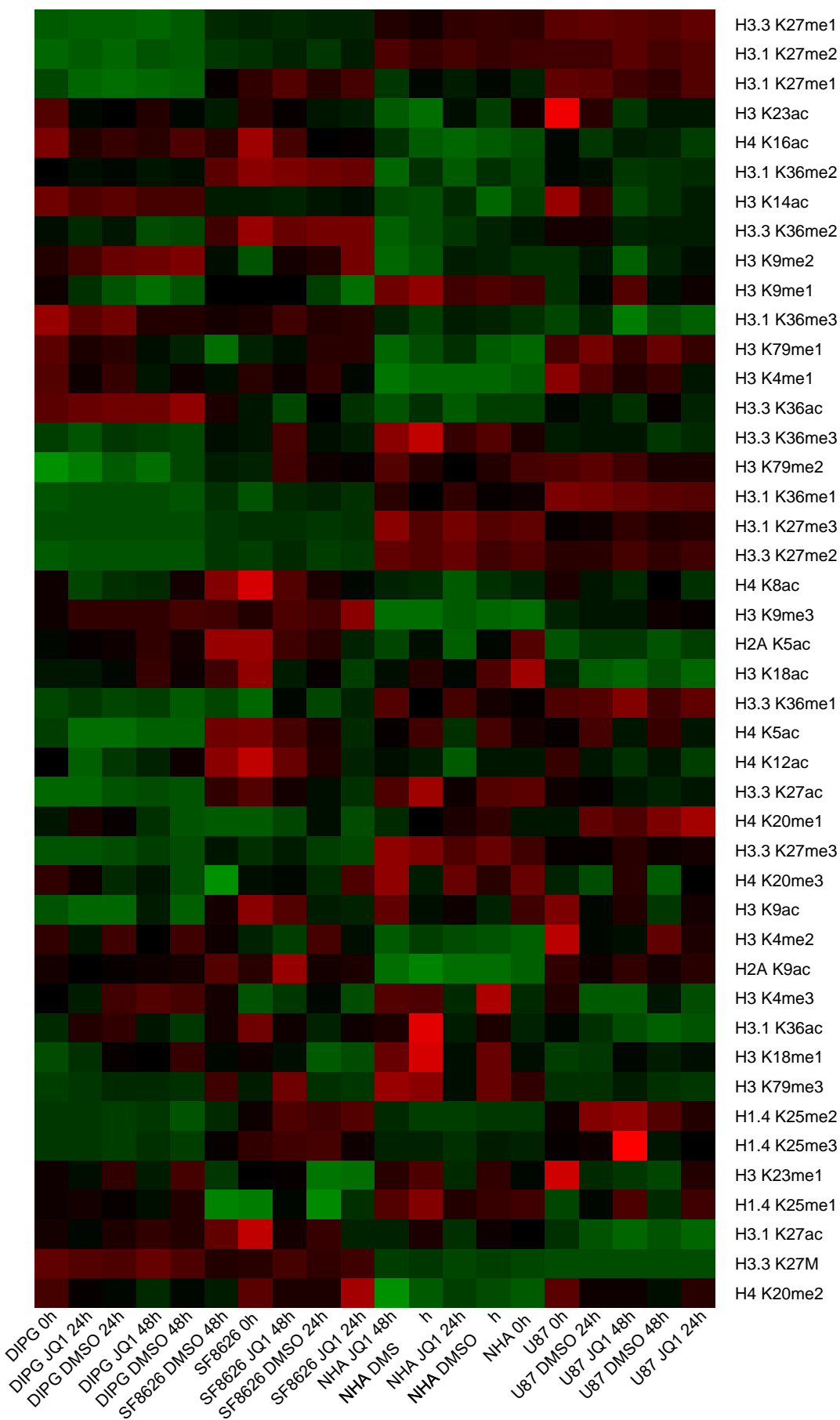

Supplement: Supplementary file 10 — Additional file 10: S10. Functional pathways analysis of differentially expressed genes after RT and Bromodomain inhibition. A Differentially expressed genes by DIPG cells after 48 h RT compared to DMSO control enrich for DNA double-strand break repair as a top canonical pathway on functional analysis (p-value 1.10 × 10− 21). B Differentially expressed genes by DIPG cells after 48 h JQ1 treatment compared to DMSO control enrich for nucleotide excision repair as the top canonical pathway (p-value 2.51 × 10− 48). [file 13046_2020_1773_MOESM10_ESM.pdf]
