# Supplementary material for: Histone tail analysis reveals H3K36me2 and H4K16ac as epigenetic signatures of diffuse intrinsic pontine glioma
Source: J Exp Clin Cancer Res. 2020 Nov 25;39:261. doi: 10.1186/s13046-020-01773-x (PMC7687710; doi:10.1186/s13046-020-01773-x)
Supplement: Supplementary file 12 — Additional file 12. [file 13046_2020_1773_MOESM12_ESM.pptx]

## Slide 1
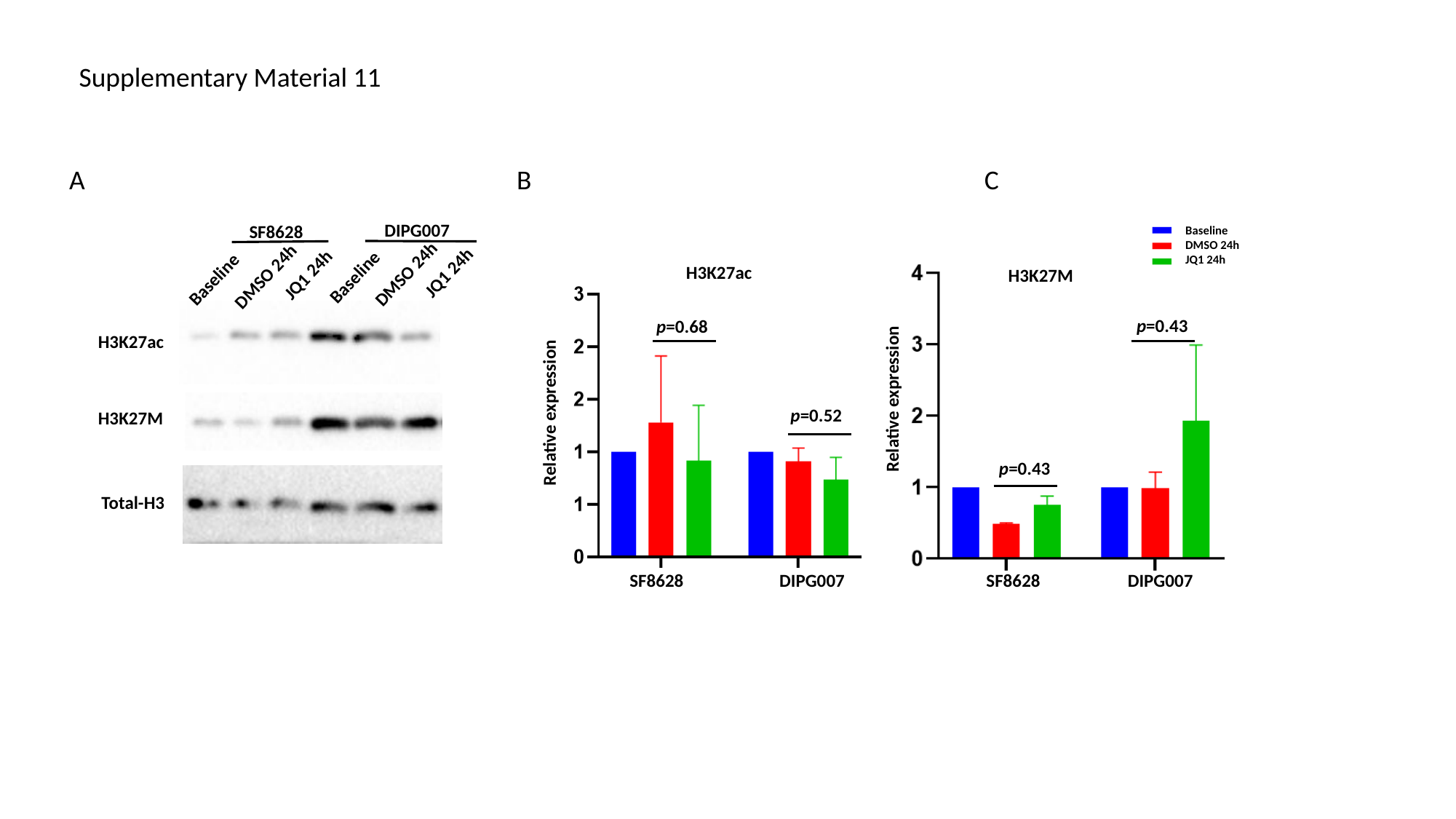

Supplementary Material 11
A
B
C
DIPG007
SF8628
Baseline
DMSO 24h
JQ1 24h
JQ1 24h
DMSO 24h
JQ1 24h
H3K27ac
Baseline
DMSO 24h
H3K27M
Baseline
p=0.43
p=0.68
H3K27ac
Relative expression
Relative expression
p=0.52
H3K27M
p=0.43
Total-H3
SF8628 DIPG007 SF8628 DIPG007
